# POF-based biosensors for cortisol detection in seawater as a tool for aquaculture systems

**DOI:** 10.1038/s41598-024-63870-7

**Published:** 2024-06-07

**Authors:** Francesco Arcadio, Simone Soares, Jan Nedoma, Dayana Aguiar, Ana Cristina Pereira, Luigi Zeni, Nunzio Cennamo, Carlos Marques

**Affiliations:** 1 Department of Engineering, University of Campania Luigi Vanvitelli, Via Roma 29, 81031 Aversa, Italy; 2 CICECO –Aveiro Institute of Materials & Physics Department, University of Aveiro, Campus Universitário de Santiago, 3810-193 Aveiro, Portugal; 3 I3N & Physics Department, University of Aveiro, Campus Universitário de Santiago, 3810-193 Aveiro, Portugal; 4 Department of Telecommunications, VSB – Technical University of Ostrava, Ostrava, 70800 Czech Republic; 5 ISOPlexis, Centre for Sustainable Agriculture and Food Technology, University of Madeira, Campus da Penteada, 9020-105 Funchal, Portugal; 6 Chemical Process Engineering and Forest Products Research Centre, Department of Chemical Engineering, University of Coimbra, Pólo II—Rua Sílvio Lima, 3030-790 Coimbra, Portugal; 7 Department of Physics, VSB – Technical University of Ostrava, Ostrava, 70800 Czech Republic

**Keywords:** Optics and photonics, Physical chemistry, Marine biology

## Abstract

A surface plasmon resonance (SPR) phenomenon implemented via D-shaped polymer optical fiber (POF) is exploited to realize cortisol biosensors. In this work, two immonosensors are designed and developed for the qualitative as well as quantitative measurement of cortisol in artificial and real samples. The performances of the POF-based biosensors in cortisol recognition are achieved using different functionalization protocols to make the same antibody receptor layer over the SPR surface via cysteamine and lipoic acid, achieving a limit of detection (LOD) of 0.8 pg/mL and 0.2 pg/mL, respectively. More specifically, the use of cysteamine or lipoic acid changes the distance between the receptor layer and the SPR surface, improving the sensitivity at low concentrations of about one order of magnitude in the configuration based on lipoic acid. The LODs of both cortisol biosensors are achieved well competitively with other sensor systems but without the need for amplification or sample treatments. In order to obtain the selectivity tests, cholesterol and testosterone were used as interfering substances. Moreover, tests in simulated seawater were performed for the same cortisol concentration range achieved in buffer solution to assess the immunosensor response to the complex matrix. Finally, the developed cortisol biosensor was used in a real seawater sample to estimate the cortisol concentration value. The gold standard method has confirmed the estimated cortisol concentration value in real seawater samples. Liquid–liquid extraction was implemented to maximize the response of cortisol in liquid chromatography coupled with tandem mass spectrometry (LC–MS/MS) analysis.

## Introduction

The population increases every year and will reach a level where fish consumption will exceed the production capacity of the oceans and seas^1^. Aquaculture can respond to this growth, as wild capture fisheries are expected to remain stable over the next years^2^.

In the last decade, aquaculture production in intensive systems has been rising rapidly, often using Recirculating Aquaculture Systems (RAS) with limited water exchange and advantages^3^. RAS has been the protein production sector with the highest growth in the world^4^, calling for high-tech progress^5^. RAS is in line with the objectives of the EU for sustainable aquaculture by producing food with a minimum ecological impact on natural resources^6^.

RAS are complex systems where fish biomass and water chemistry/quality interact. Small variations may result in sub-optimal conditions, which can induce stress, leading to a reduced food intake, consequently to ingrowth and in more extreme cases to mortality^7^. Fish has one of the best conversion ratios of feed to meat among all animals, and aquaculture is the fastest-growing industry in food animal production. However, when the ideal environment is not ensured, the conversion ratio drastically drops, and fish well-being is compromised. Therefore, stress affects the growth and feeding of fish, thus feed conversion ratio drops, leading to high costs and production loss.

As in humans, fish have a similar pathway to respond to stress. It starts in the hypothalamus where the corticotropin hormones are produced. Then, these hormones will stimulate the adrenocorticotropic hormone (ACTH) release in the corticotroph cells of the anterior pituitary. Subsequently, the ACTH will lead to the release of corticosteroids in the blood vessels by inter-renal cells (adrenal cortex homolog)^8^. The corticosteroids can then passively diffuse to cells or could be diffused out of the fish, to the water, through the gills^9^. In most types of fish, cortisol is the principal produced corticosteroid and influences several mechanisms such as neurogenesis, growth, and reproductive and immune systems. One study has searched the levels of cortisol of four species of fish (rainbow trout, gilthead sea bream, Senegalese sole, and sea bass) when they were controlled and in stress^10^. In control, the cortisol levels in fish blood are between 5.65–26.3 ng/mL, and when in stress the levels of cortisol are between 24.2–114.6 ng/mL.

The traditional way to measure cortisol in fish is through blood sampling and analysis of blood plasma: an invasive method whose results are influenced by the sampling method itself. Also, it is time-consuming, and the results are not immediate. It was demonstrated that the cortisol release rate into the water of European sea bass increased in response to stress and was correlated with plasma cortisol concentrations from the blood (at low concentrations, ~ 0.007 ng/mL)^11^. Therefore, cortisol can be monitored by analyzing the water from the fish tanks. One recent study has shown that these levels decrease when cortisol is analyzed through water samples from fish tanks, between 1 and 3 ng/mL in normal conditions but can also vary considerably more in other environments^12^.

Consequently, it is essential to monitor fish stress mitigating the negative consequences. This is one of the industry's major concerns that needs to be addressed to enhance aquaculture technological progress. Such a challenge can be answered by exploiting innovative technology. One possibility that has been researched is the utilization of cortisol as a biomarker in biosensors for point of care (POC) since cortisol proved to be a reliable indicator of stress, even in fish. Hence, biosensors capable of detecting cortisol on POC have been explored in marine biology, particularly in aquaculture, to monitor stress levels in fish. Optical biosensors are one of the different strategies that have been employed in cortisol biosensing. Compared to silica fibers, polymeric optical fibers (POFs) are attractive for application in this industry due to their flexibility, high numerical aperture, easiness of handling, and associated low-cost instrumentation^13^. Besides, surface plasmon resonance (SPR) is widely used in biosensing due to sensitivity enhancement. This phenomenon occurs in the presence of a metal–dielectric interface. Therefore, evanescent waves (EWs), created during total internal reflection (TIR) of light in the optical fiber, excite the electrons of the metallic surface creating quantized collective oscillations of free electrons, which are categorized as surface plasmons (SPs). When these free electrons collectively oscillate in a metal, occurs the creation of p-polarized electromagnetic waves, namely surface plasmon waves (SPWs). SPR phenomenon is triggered when p-polarized light hits the interface and if the propagation constant and energy of the resultant EWs are equal to those of the SPWs. When this phenomenon occurs, a strong absorption of light is created, and the sensor spectrum shows a sharp dip for a specific wavelength called resonance wavelength, which is highly sensitive to changes in the surrounding refractive index (RI), so these variations can be determined by the analysis of resonance wavelength^14^.

Oppositely to prism-based configurations, which are usually bulky and require expensive optical components, the use of optical fibers to build SPR sensors is known to improve plasmonic performance, making it possible to reduce the device’s cost and dimensions hence paving the way to the use outside the laboratory scenario. To this purpose, SPR biosensors based on optical fibers (i.e., POFs) have proved to be an advantageous solution to realize ultra-sensitive platforms mainly because of the easiness in the fabrication steps required to modify the optical fiber to attain the sensitive area, such as in the case of D-shaped POFs, tapered POFs, and so on^15^.

This work aims to develop and test the feasibility of a D-shaped POF immunosensor for cortisol detection, using the SPR effect, promoted by gold film. In this work, two sensors with different functionalizations were tested. Regarding the first steps, in one case was used cysteamine and in the other lipoic acid, however, both were functionalized with the same anti-cortisol antibodies. In particular, cysteamine and lipoic acid were utilized due to the enhancement of their anti-fouling capability.

Preliminary cortisol detection tests were performed using different cortisol concentrations in PBS, ranging from 0.0001 to 0.1 ng/mL, for the two immunosensors subject to different functionalizations, achieving LODs of 0.0008 ng/mL and 0.0002 ng/mL when using cysteamine and lipoic acid, respectively. Moreover, control tests for selectivity assessment were carried out using cholesterol and testosterone as the interfering substances in a sensor functionalized with anti-cortisol antibodies. Furthermore, a test in simulated seawater diluted 1:50 was performed for the same cortisol concentration range, to assess the immunosensor response to complex matrix. Finally, this immunosensor was also tested in real seawater samples and the obtained results were then compared with those achieved by a standard analytical method.

These immunosensors are mainly developed for future application in the aquaculture sector, specifically in RAS fish tanks for in situ and real-time detection of cortisol levels present in fish water in order to mitigate the stress induced due to the complex RAS understanding the dynamics of entire RAS, correlate it with other measured parameters in fish water as well as consider such parameter for predictive measures. However, these sensors can also be applied in other critical areas of intrinsic human health to detect cortisol levels in blood or sweat samples to mitigate certain diseases. Such biosensors can contribute to a better understanding of such complex RAS to produce quality food and improve human nutrition.

## Results and discussion

### Preliminary binding tests in PBS

**Figure 1:**
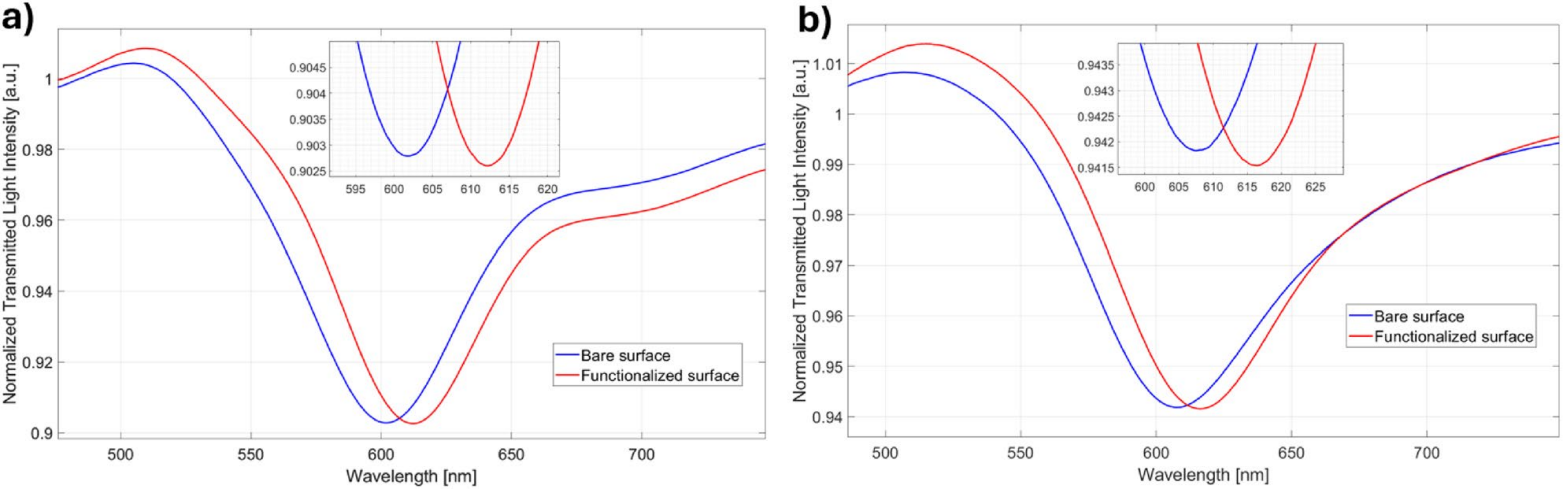
SPR spectra obtained before (blue line) and after (red line) two different functionalization procedures based on (**a**) lipoic acid and (**b**) cysteamine.

Two different functionalization protocols were explored for cortisol detection, i.e., one based on lipoic acid and another one based on cysteamine, as described in Methods Section. It is important to underline that, in the view of comparing the two biosensor configurations, the same antibody concentration and EDC/NHS molar ratio were used, apart from the thiol ligand. In order to assess the effectiveness of both the functionalization protocols, it was evaluated the change in resonance wavelength produced with respect to the bare platforms and by considering the same bulk solution, i.e. PBS. As it is clear from the SPR spectra reported in Fig. 1, both configurations (based on lipoic acid and cysteamine) denoted an increase in the resonance wavelength (red-shift) of about 10 nm following the functionalization procedure. These results testify that the SAMs were successfully formed on the gold film. In fact, when the receptor layer is bonded to the gold surface, the measured refractive index value increases with respect to the bare surface (i.e., without receptor) when the same bulk solution (i.e., PBS) is used.

Next, in order to determine the SPR-POF immunosensors response to cortisol and to compare the biosensing performance achieved by the immunsosensors functionalized with two different functionalization protocols, both the configurations (based on lipoic acid and cysteamine) were challenged with cortisol solution in PBS at different concentrations ranging from 0.0001 to 0.1 ng/mL. Preliminarily, in order to optimize the incubation time, the cortisol-anti-cortisol antibody binding kinetics for both the configurations were obtained (see Figure S1 in Supplementary Information file) for a cortisol concentration of 0.05 ng/mL, and by acquiring the SPR spectra each 2 min, for twelve minutes. As shown in Figure S1, 10 min can be used to achieve the receptor-analyte binding in both configurations.

**Figure 2:**
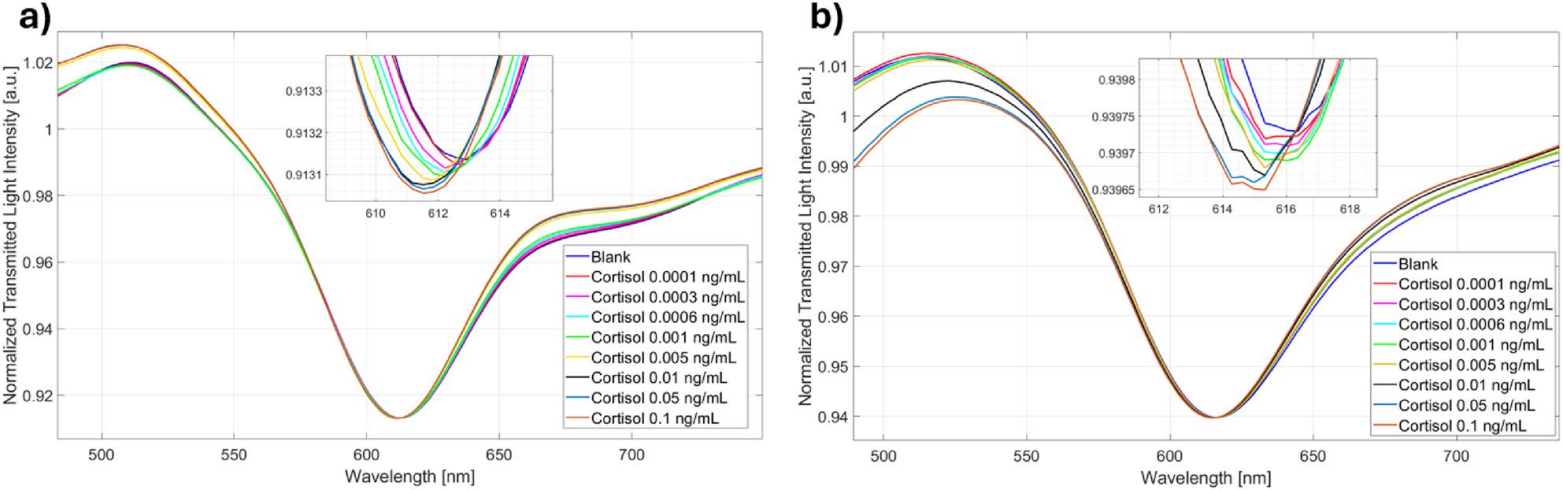
SPR spectra obtained at different cortisol concentrations in PBS (0–0.1 ng/mL) on two immunosensor configurations based on (**a**) lipoic acid and (**b**) cysteamine.

Figure 2 shows the SPR spectra attained by the two immunosensor configurations for several cortisol concentrations and by following the measuring protocol detailed in Methods Section. In both cases, the binding between cortisol and receptor layer produced a shift toward lower values of the resonance wavelength, so indicating that the refractive index of the receptor layer on the gold surface decreased after the binding with the analyte. This behaviour was similar to those achieved by the same SPR-POF probe coupled with other receptors, and it is typically related to a modification in the conformation of the receptor itself^16–18^.

**Figure 3:**
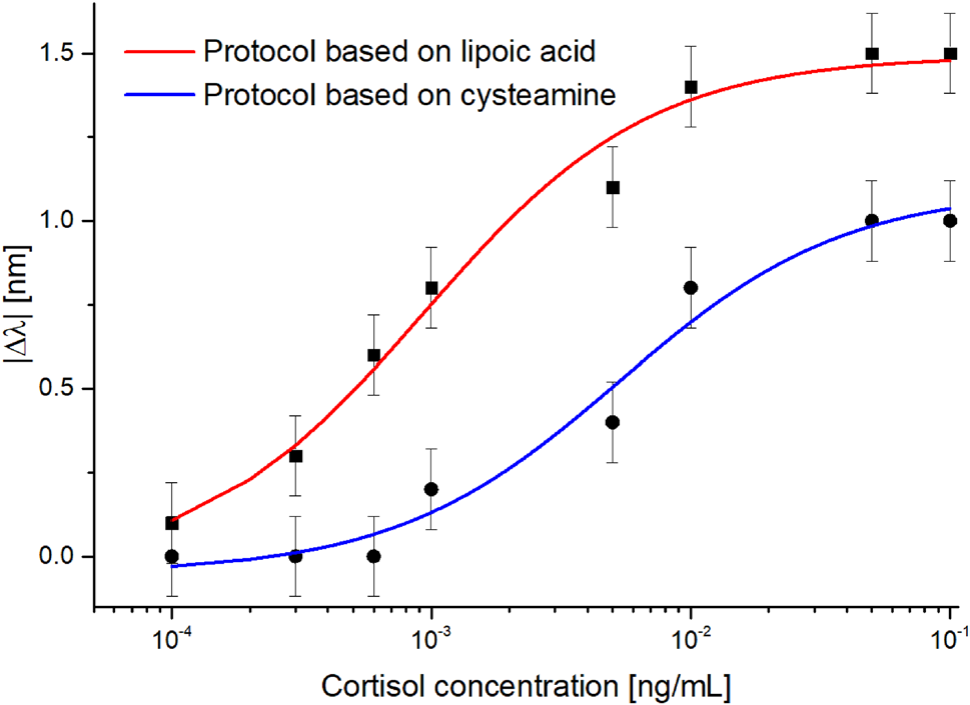
Absolute value of the resonance wavelength shift as a function of cortisol and Langmuir fittings of the experimental values relative to two immunosensor configurations based on lipoic acid (red line and black square markers) and cysteamine (blue line and black circle markers).

Figure 3 shows the dose–response curves, in terms of absolute value of resonance wavelength variations (|∆λ|), computed with respect to the solution without cortisol (blank solution) as a function of the cortisol concentration, in a semilog scale, for both the immunsensor configurations. Furthermore, the Langmuir fittings of the experimental values and the error bars are also reported in Fig. 3 for the configuration based on lipoic acid (red line and black square markers) and cysteamine (blue line and black circle markers). The error bars, for each configuration, were calculated as the maximum standard deviation obtained in three different measurements and resulted equal to 0.12 nm. The Langmuir fitting parameters for both configurations are listed in Table 1.

**Table 1:** Langmuir fitting parameters relative to cortisol detection in PBS by two different immunosensors based on two functionalization procedures.

Cortisol immunosensor configuration	λ_0_ (nm)		Δλ_max_ (nm)		K (ng/mL)		Statistics	
	Value	St. error	Value	St. error	Value	St. error	χ^2^	R^2^
Based on lipoic acid	− 0.0403	0.1050	1.4923	0.0499	0.0009	0.0002	0.0062	0.9793
Based on cysteamine	− 0.0508	0.0559	1.0943	0.0761	0.0053	0.0017	0.4606	0.9667

The Langmuir equation is a commonly adopted model to describe specific antibody/analyte interactions where a saturation phenomenon is present, like in the case presented here, because of the finite number of receptors bonded on the gold sensitive area^19,20^. The related fitting parameters (listed in Table 1) allow for calculating the biosensing performance parameters in terms of sensitivity at low concentration (S_low c_), limit of detection (LOD), and affinity constant (previously defined as the reciprocal of K parameter). More specifically, the sensitivity at low concentration is calculated in the hypothesis that c $\ll$ K, being the fact that in this case Eq. (1) can be considered linear and S_low c_ represents the slope (equal to $\left|{\Delta \lambda }_{max}/K\right|$) of this linear function. On the other hand, the LOD is computed as the ratio of three times the standard deviation of the blank (St. error of λ_0_ in Table 1) and the S_low c_^21^. The biosensing performance parameters above defined are reported in Table 2 for both the cortisol immunosensor configurations based on lipoic acid and cysteamine. As it is clear from Table 2, the cortisol biosensor harnessing the functionalization procedure based on lipoic acid can be preferred because it denotes overall better performance parameters with respect to the other configuration based on cysteamine.

**Table 2:** Analytical parameters relative to cortisol detection in PBS achieved by two different immunosensors based on two functionalization procedures.

Cortisol immunosensor configuration	Sensitivity at low concentration ($\left|{\Delta \lambda }_{max}/K\right|$)	LOD ((3 $\times$ St. Error (λ_0_))/S_low c_)	K_aff_ (1/K)
Based on lipoic acid	1587.6 nm mL ng^−1^	0.0002 ng/mL	1063.8 mL/ng
Based on cysteamine	206.1 nm mL ng^−1^	0.0008 ng/mL	188.3 mL/ng

### Selectivity test: interfering substances

**Figure 4:**
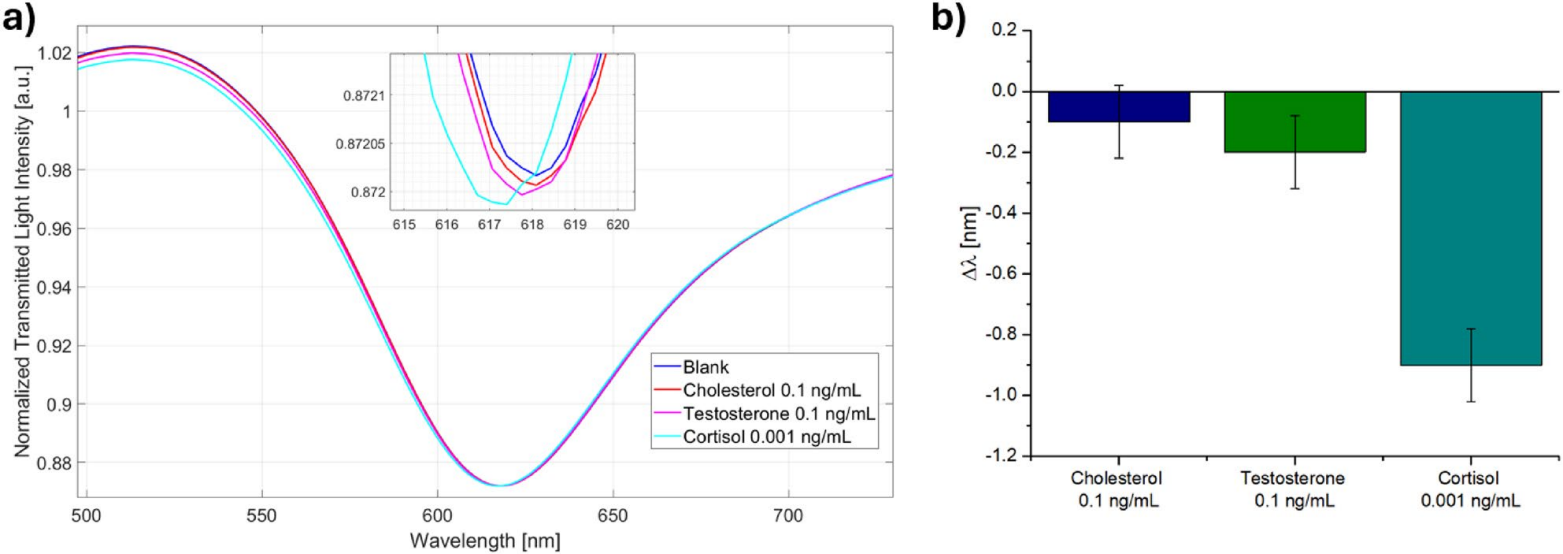
(**a**) SPR spectra and (**b**) resonance wavelength variation (∆λ) relative to the interferents (cholesterol and testosterone) at 0.1 ng/mL and the analyte (cortisol) at 0.001 ng/mL.

The selectivity of the proposed cortisol immunosensor was assessed by challenging the optimized configuration (based on lipoic acid) with possible interfering substances, meaning cholesterol and testosterone. The results summarized in Fig. 4 highlight that the cortisol immunosensor response is not affected by the tested interfering substances. In fact, the resonance wavelength variations obtained by cholesterol and testosterone (both at a concentration of 0.1 ng/mL), were much lower (within the error bars) with respect to the one of the cortisol, which was tested at a concentration one hundred times lower (0.001 ng/mL).

### Test in simulated seawater diluted 1:50

The response of the optimized cortisol immunosensor (based on lipoic acid) was evaluated in simulated seawater solution diluted 1:50, as described in Methods Section. The tested cortisol concentration range was the same as for the preliminary binding experiments in PBS.

**Figure 5:**
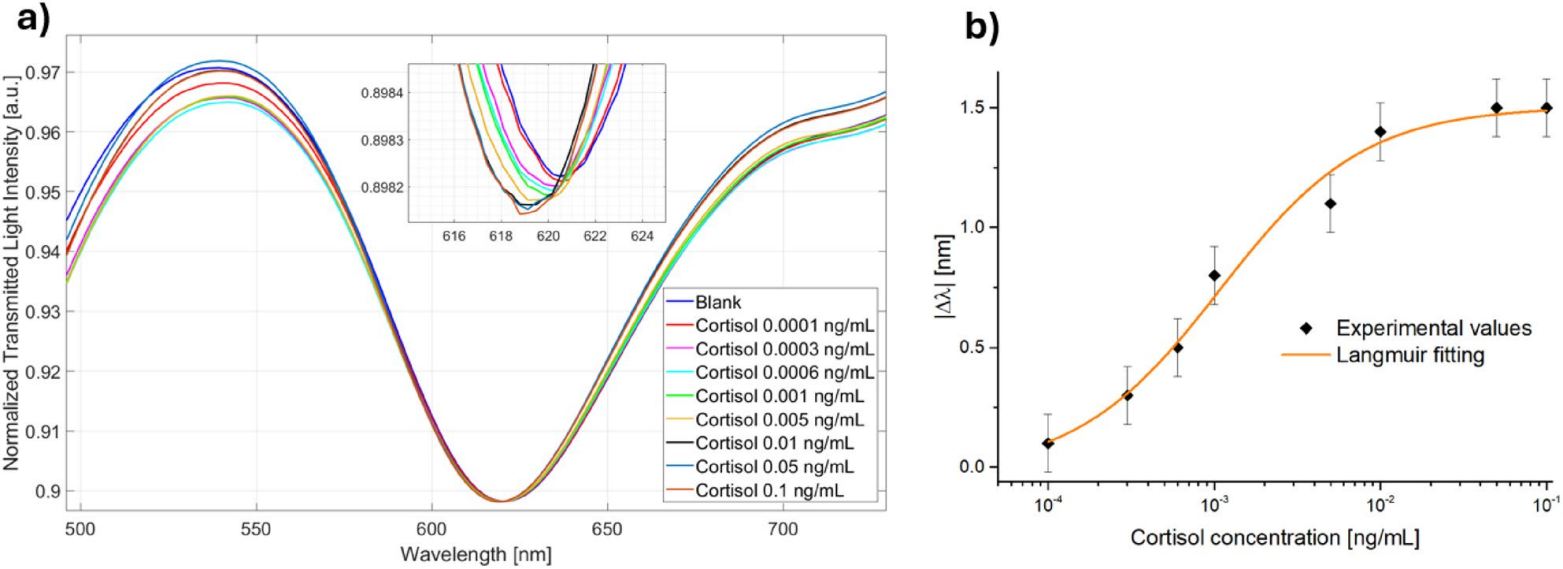
(**a**) SPR spectra obtained at different cortisol concentrations (0–0.1 ng/mL) and (**b**) dose/response curve achieved in simulated seawater diluted 1:50 on the immunosensor configuration based on lipoic acid.

Figure 5a reports the SPR spectra achieved by testing the immunosensor based on lipoi acid with cortisol solutions in a concentration range between 0.0001 ng/mL and 0.1 ng/mL, whereas Fig. 5b reports the dose/response curve obtained by the Langmuir fitting (whose parameters are reported in Table 3) of the experimental values. As it can be stated from the biosensing parameters reported in Table 4, the experimental results achieved in simulated seawater diluted 1:50 were very similar and in line with those obtained by the same immunosensor configuration in the preliminary binding tests in PBS. Thus, it is reasonable to state that the proposed immusensors response is not significantly influenced by the simulated seawater matrix, being highly specific for cortisol.

**Table 3:** Langmuir fitting parameters relative to cortisol detection in simulated seawater diluted 1:50 by the immunosensor configuration based on lipoic acid.

λ_0_ (nm)		Δλ_max_ (nm)		K (ng/mL)		Statistics	
Value	St. error	Value	St. error	Value	St. error	χ^2^	R^2^
− 0.0230	0.0957	1.5050	0.0495	0.0011	0.0003	0.4041	0.9813

**Table 4:** Analytical parameters relative to cortisol detection in two different matrices achieved by the immunosensor configuration based on lipoic acid.

Matrix	Sensitivity at low concentration ($\left|{\Delta \lambda }_{max}/K\right|$)	LOD (3 $\times$ St. Error (λ_0_)/S_low c_)	K_aff_ (1/K)
PBS	1587.6 nm mL ng^−1^	0.0002 ng/mL	1063.8 mL/ng
Simulated seawater diluted 1:50	1414.9 nm mL ng^−1^	0.0002 ng/mL	925.9 mL/ng

For the sake of comparison, Table 5 reports a comparative analysis in terms of LOD between several sensors for cortisol detection in several matrices presented in the literature and based on different sensing approaches.

**Table 5:** Comparison in terms of detection limit between several cortisol sensors in different matrices.

Transducer	Receptor	Sensing approach	LOD	Matrix	Reference
3D origami microfluidic chip	Aptamer	Fluorescence	6.76 ng/mL	Artificial sweat	^22^
Lateral flow immunoassay (LFIA) integrated in a smartphone	Antibody	Chemiluminescence	0.3 ng/mL	Saliva	^23^
poly(dimethylsiloxane) (PDMS) microfluidic coupled with metal–oxide–semiconductor (CMOS) optical detection system	Antibody	Colorimetric	18 pg/mL	Buffer solution	^24^
Electrode covered with NiO thin film	Antibody	Electrochemical	0.32 pg/mL	PBS	^25^
Optical fiber based on laser-induced graphene	Antibody	Interferometry	0.1 ng/mL	PBS	^26^
gold nanoparticle (AuNP) layers deposited on functionalized poly (dimethylsiloxane) (PDMS) substrate	Aptamer	LSPR	36.2 pg/mL	Sweat	^27^
S-flex fiber optic coupled with gold nanoparticles	Antibody	LSPR	147.9 pg/mL	PBS	^28^
stretchable PDMS base with carbon nanotubes-cellulose nanocrystals (CNC/CNT) conductive nanoporous nanofilms	Molecularly imprinted polymer (MIP)	Electrochemical	2 ng/mL	Sweat	^29^
Polarization-based nanoplatform	nanoMIPs	Fluorescence	0.28 ng/mL	chloroform/ hexane (4:1, v/v)	^30^
Nitrogen doped bamboo-liked carbon nanotubes loaded with nickel nanoclusters	Electropolymerized MIP	Electrochemical	8.6 fg/mL	Saliva	^31^
SPR-POF probe coupled with anti-cortisol antibody (protocol based on cysteamine)	Antibody	SPR	0.8 pg/mL	PBS	This work
SPR-POF probe coupled with anti-cortisol antibody (protocol based on lipoic acid)	Antibody	SPR	0.2 pg/mL	PBS/Simulated seawater	This work

### Test *in real* seawater samples

**Figure 6:**
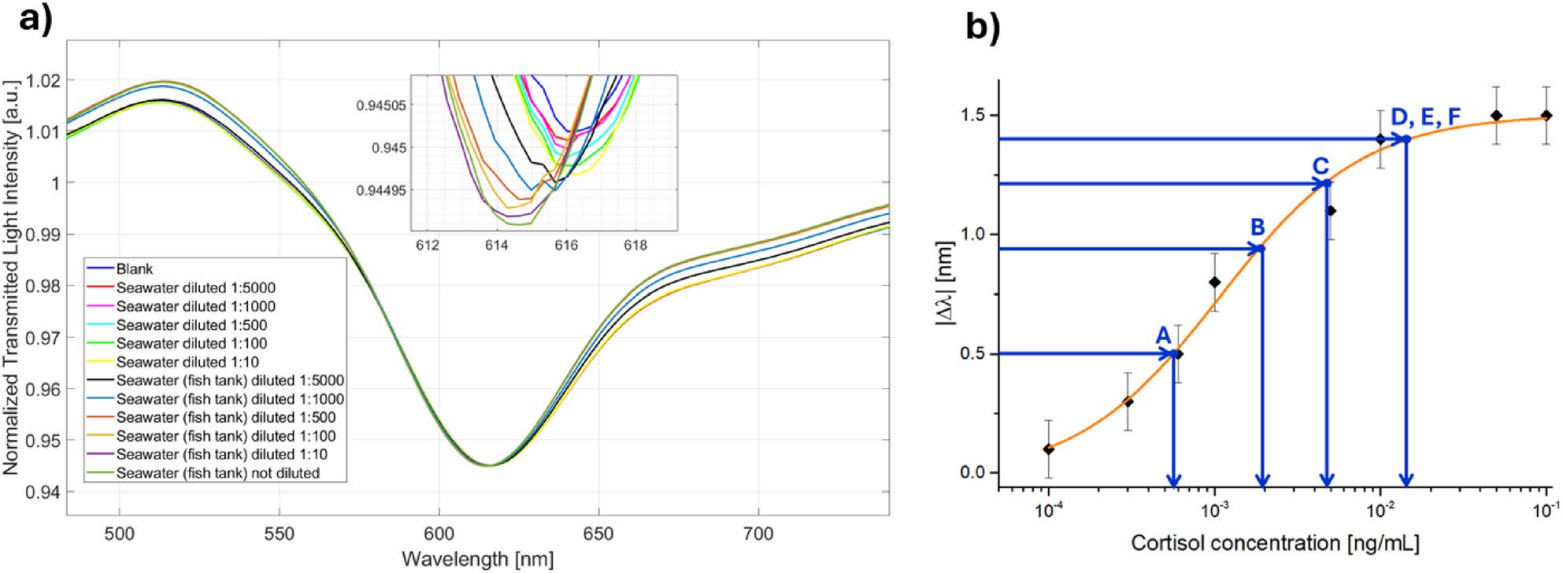
(**a**) SPR spectra relative to cortisol detection in two seawater samples collected from two different tanks (with and without fish), and diluted with PBS at different dilution factors. (**b**) Cortisol concentration estimation from the binding isotherm achieved in simulated seawater diluted 1:50.

As a proof of concept, in order to assess the applicability of the proposed cortisol immunosensor in a real scenario, further tests were carried out with real seawater samples. More specifically, two seawater samples were collected, one from a tank without fish and another one from a tank with fish, where the cortisol presence is expected. Both the samples were diluted and prepared as described in Methods Section. From the SPR spectra shown in Fig. 6a, it can be observed a blueshift only in relation to the sample collected from the tank with fish, and diluted at different dilution factors. In fact, no cortisol was detected from the seawater samples collected from the tank without fish.

The cortisol concentration in the seawater sample collected from the tank with fish can be estimated by the resonance wavelength shifts achieved at different dilution factors and the dose/response curve reported in Fig. 5b, as shown in Fig. 6b. In particular, by considering Fig. 6b, each resonance wavelength shift (achieved at different dilutions) indicated on the y-axis by blue lines can be used to estimate the corresponding cortisol concentration on the x-axis. To this purpose, Table 6 summarizes the resonance wavelength shifts (|Δλ|) obtained at different dilution factors and the corresponding cortisol concentration attained by the binding isotherm in simulated seawater diluted 1:50. The cortisol concentration in the seawater sample can be estimated by multiplying the concentration reported in Table 6 with the related dilution factor. More specifically, from the A, B and C experimental data (see Table 6), the cortisol concentration in the analyzed seawater sample is around 2.5–2.7 ng/mL. Oppositely, D,E, and F are only useful for an estimation of the lower bound of the concentration since these values fell in the saturation area of the cortisol immunosensor.

**Table 6:** Summary of the SPR biosensor response of the seawater sample from fish tank tested at different dilution factor ad estimation of the cortisol concentration from the binding isotherm.

Label	Dilution factor	∆λ (nm)	Estimated cortisol concentration of the diluted seawater sample (ng/mL)	Estimated cortisol concentration of the seawater sample (ng/mL)
A	1:5000	0.5	5.4 $\times$ 10^–4^	(5.4 $\times$ 10^–4^)*(5 $\times$ 10^3^) = 2.7
B	1:1000	0.9	2.5 $\times$ 10^–3^	(2.5 $\times$ 10^–3^)*(1 $\times$ 10^3^) = 2.5
C	1:500	1.2	5 $\times$ 10^–3^	(5 $\times$ 10^–3^)*(5 $\times$ 10^2^) = 2.5
D	1:100	1.4	1.5 $\times$ 10^–2^	–
E	1:10	1.4	1.5 $\times$ 10^–2^	–
F	Not diluted	1.4	1.5 $\times$ 10^–2^	–

Moreover, in order to determine the cortisol concentration of the aquaculture water samples by a gold standard technique, an analytical method based on liquid–liquid extraction was implemented to maximize the response of cortisol in liquid chromatography coupled to tandem mass spectrometry (LC–MS/MS) analysis.

Cortisol levels were first determined in water samples collected from four different points of the RAS but for this work only two points were considered: in "control water" and "water collected after contact with fish". From the analysis of the results, the highest cortisol concentration determined was in the "water collected after contact with fish" sample, with approximately 2.10 ng/mL. Regarding "control water" sample, it is the water treatment with the biofilter, which we consider the water with very reduced cortisol concentration. Table 7 shows the results of the analytical method used to achieve the cortisol concentration in two samples: "control water" and "water after contact with fish".

**Table 7:** Cortisol concentration in water samples from Flounder aquaculture. Concentration is expressed as mean value ± standard deviation. Different letters represent statistically significant differences (*p* < 0.05).

Sample	Cortisol (ng/mL)
Control water	0.21 ± 0.00^A^
Water after contact with fish	2.09 ± 0.06^B^

The results are in good agreement with the experimental results listed in Table 6. In particular, the recovery of the measured sample resulted to be around 120%, thus denoting a slight matrix effect.

### Reproducibility, repeatability and reusability of the cortisol biosensors

The reproducibility and repeatability of the proposed cortisol biosensors were evaluated by carrying out different experimental tests. More in detail, for each biosensor configuration, three different probes were derivatized as described in Methods Section and tested in the same cortisol concentration range (0–0.1 ng/mL) and the same conditions. The maximum value of the obtained standard deviations is used as error bars in Figs. 3 and 5b.

On the other hand, the repeatability and reusability of the cortisol biosensors are related to the effectiveness of the regeneration process, which is performed by glycine/HCl (10 mM, pH 2.2) incubations and subsequent storage in PBS at 4 °C. It should be stressed that this regeneration procedure is based on a well-established protocol^17^, and its efficiency was demonstrated by considering the experimental tests achieved in three different regeneration/binding test cycles over one month. Over this period, the sensor response, intended as the resonance wavelength variation normalized to the maximal response attained at first, resulted in a fully maintained result.

The main limitations of the proposed cortisol biosensors are related to the antibody cost and the relatively long time required for the functionalization process (about 2 days). Additionally, proper storage conditions are required, and attention should be taken into using real samples, which could lead to antibody denaturation. To overcome these drawbacks, replacing the antibody with a synthetic receptor, e.g., molecularly imprinted polymers (MIPs) could be beneficial.

## Conclusions

An SPR platform based on D-shaped POFs has been coupled with a specific antibody in order to achieve cortisol sensors. More specifically, the distance between the receptor layer and the SPR gold surface has been changed by exploiting cysteamine and lipoic acid in the functionalization process. The best biosensor configuration is obtained by exploiting the lipoic acid, improving the sensitivity at low concentrations of about one order of magnitude. Moreover, the LOD value is about 0.2 pg/mL instead of 0.8 pg/mL achieved by the cysteamine configuration. The best cortisol biosensor configuration has been tested with cholesterol and testosterone used as interfering substances. In order to obtain the sensor's calibration curve, the biosensor has been tested in simulated seawater for the same cortisol concentration range achieved in buffer solution. Finally, the developed cortisol biosensor has been used to estimate the cortisol concentration value of a real seawater sample. The obtained cortisol concentration value has been confirmed via a gold standard method. From the experiment results, the proposed POF-based biosensor could be considered a simple and low-cost tool useful in aquaculture systems for measuring cortisol concentrations in seawater.

## Methods

### Chemicals

N-(3-dimethylaminopropyl)-N'-ethylcarbodiimide hydrochloride (EDC), N-hydroxysuccinimide (NHS, 98%), and lipoic acid (> 99%) were purchased from Merck, Germany. Bovin serum albumin (BSA), phosphate buffered saline (PBS) tablets (pH = 7.4), and ethanol absolute (≥ 99.8%) were obtained from Fisher Bioreagents, USA. Deionized (DI) water was obtained from a Milli-Q water purification system and was used as received. Cysteamine hydrochloride (≥ 98%), hydrocortisone (cortisol, ≥ 98%), cholesterol (≥ 99%), and 17α-Methyltestosterone (testosterone, ≥ 97%) were purchased from Sigma-Aldrich, Germany. The cortisol polyclonal antibody (5 mg/mL) was acquired from Invitrogen, EUA.

### Functionalization protocols

The functionalization of the SPR-POFs is a crucial step to obtain cortisol responsiveness. Therefore, anti-cortisol antibodies were immobilized onto the surface of the fibers acting as the biorecognition element, allowing the binding of cortisol. In this work, we compare two functionalization procedures. Before functionalization, the POF surfaces were cleaned three times with DI water.

In one functionalization, due to the strong physicochemical interaction of Au and sulfur, was used a thiol derivative, cysteamine. The latter is a thiol derivative, allowing a strong physicochemical interaction of sulfur and Au, and the creation of amine groups (NH_2_) on the surface for the immobilization of the antibodies. In this way, the COO^-^ functional groups of the antibody covalently bind to the amine groups of cysteamine. In this way, this functionalization started with the incubation of a POF overnight at room temperature in an aqueous solution of cysteamine (20 mM), allowing the creation of amine groups on the surface. Thereafter, POF was washed with DI water and PBS (three times each) to remove unbounded cysteamine. Next, it was placed, for 2 h at room temperature, a 15 µL of anti-cortisol antibody (0.88 mg/mL), 50 μL of EDC (0.5 M, in PBS) and 50 μL of NHS (0.2 M, in PBS). The antibodies were covalently immobilized through the carboxylic acid functional groups to the functional groups of cysteamine in the fiber surface, using EDC/NHS bioconjugate reagents. EDC activates carboxylic groups promoting the formation of amide bonds when in the presence of amine groups, a reaction that can be enhanced by using NHS. Afterwards, surfaces were washed three times with PBS to eliminate the excess of the antibody.

The other functionalization started with the incubation of POF overnight at room temperature with lipoic acid at a final concentration of 0.3 mM in 8% ethanol solution, allowing the creation of carboxyl groups on the surface. This reagent contains both a carboxyl group and a dithiol group, which strongly binds to the Au surface after reduction. Thereafter, POF was washed with DI water and PBS (three times each). For the subsequent activation of the carboxyl groups, a solution of EDC/NHS (0.5 M/0.2 M in PBS) was placed in contact with the POF surface for 20 min at room temperature. Afterwards, surfaces were washed three times with PBS to eliminate the excess of the used reagents. The next step was the incubation for 2h at room temperature with 15 µL of anti-cortisol antibody (0.88 mg/mL) for covalent immobilization. Then, the antibody excess was removed by three washes with PBS.

After the antibody immobilization in the two functionalization procedures, the POF surfaces were passivated with BSA (5 mg/mL) for 30 min at room temperature. Finally, the washing step was repeated three times with PBS, and the POFs were stored in PBS overnight at 4 °C and finally washed with PBS before use.

### SPR-POF sensor platform

The SPR-POF probe fabrication steps are comprehensively termed in^13^. In summary, a 1 mm POF (980 μm of core made of Poly(methyl methacrylate) and 10 μm of fluorinated cladding) was embedded into a resin support and subsequently polished by two lapping papers (5 μm and 1 μm grits, respectively) in order to achieve the D-shaped area. Next, a photoresist buffer layer (Microposit S1813, Chimie Tech Services, Antony, France), having a refractive index equal to about 1.6, was deposited on the D-shaped region by means of a spin coater. This intermediate layer was useful to improve the plasmonic performance and to improve the ensuing gold film adhesion^13^. In the end, a 60 nm thick gold film was deposited by a sputter coater machine (CCU-010, Safematic, Zizers, Switzerland). The gold film deposition was carried out at a pressure of 10^–2^ mbar, a current of 60 mA and in three consecutive steps (each of which last 20 s), in order to prevent the deposition chamber to reach high temperature.

By considering that the resonance wavelength at a fixed refractive index bulk (i.e., water as the surrounding medium) is a function of both the gold nanofilm thickness^32^ and the waveguide characteristics (D-shaped POF regions)^33^, the SPR wavelength value in water can be used as a quality control. In particular, only the probes with an SPR wavelength in water of 600 nm+/− 3 nm are used.

### Experimental setup

**Figure 7:**
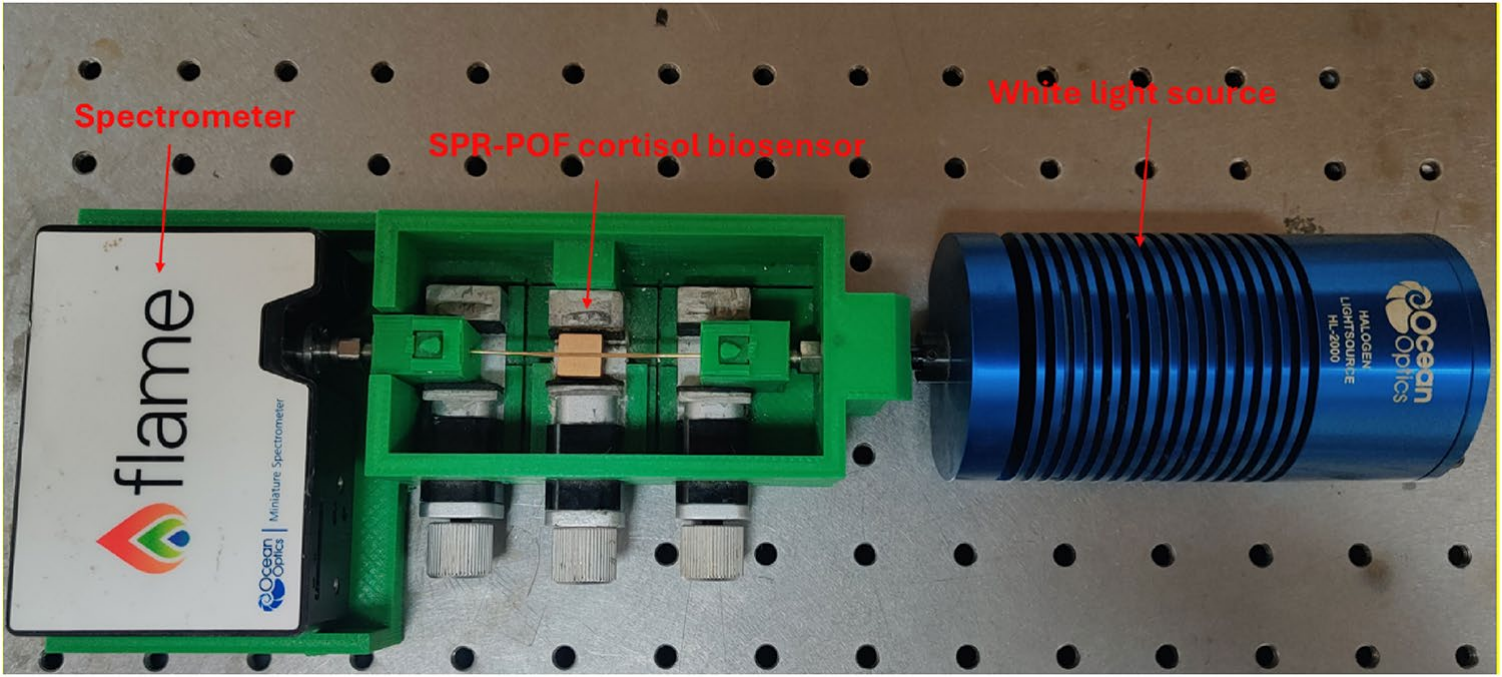
Actual picture of the experimental setup used to test the SPR-POF immunosensor in cortisol detection.

The experimental setup used to carry out the experimental tests, shown in Fig. 7, is composed by a broadband light source and a spectrometer. More specifically, a halogen lamp (HL-2000LL, Ocean Insight, Orlando, FL, USA) having an emission range between 360 and 1700 nm was used as source; on the other hand, a spectrometer (FLAME-S-VIS–NIR-ES, Ocean Insight, Orlando, FL, USA) having a detection range from 350 to 1000 nm was used as receiver. The SPR-POF immunosensor was connected with the light source and the spectrometer via SMA connectors. Finally, the spectrometer was connected by an USB cable to a laptop for the data processing.

### Measuring protocol and sample preparation

An additive immunoassay, obtained by gradually increasing the cortisol concentration, was carried out. In particular, for the preliminary binding tests in PBS, eight cortisol concentrations were used: 0.0001, 0.0003, 0.0006, 0.001, 0.005, 0.01, 0.05, and 0.1 ng/mL. These concentrations were prepared through consecutive dilutions from a stock solution using PBS. Therefore, a stock solution (1 mg/10 mL) was prepared, in which cortisol was diluted in 1% of ethanol and 99% of PBS. The measuring protocol consisted of dropping in the functionalized area of the POF immunosensor each cortisol concentration for 10 min. The incubation times were defined according to the preliminary binding kinetics information (data reported in Figure S1 of the Supplementary Information file). Between each concentration, a PBS washing step was carried out three times and the spectrum was acquired at the fourth time by considering PBS as the bulk solution. PBS was considered the blank solution.

The shift in SPR resonance wavelength (calculated with respect to the blank) as a function of the cortisol concentration was plotted.

In order to evaluate the performance of the sensors in terms of selectivity to cortisol, control tests were carried out using cholesterol and testosterone. Therefore, the POF sensor was also functionalized with anti-cortisol antibodies (following the second functionalization protocol) and tested for 0.1 ng/mL of cholesterol, 0.1 ng/mL of testosterone, and 0.001 ng/mL of cortisol. The measuring protocol was performed as carried out at the preliminary tests.

A similar test was also carried out by using the same cortisol concentrations, in simulated seawater instead of PBS. The simulated seawater was prepared by diluting 50 times with DI water a solution with 0.46 M of NaCl. In particular, the simulated seawater was chosen in order to be completely confident that the matrix was free of cortisol.

Data were fitted by Langmuir equation which is obtained in the following Hill equation (Eq. 1) by considering n = 1:

1 $$\left|{\Delta \lambda }_{c}\right|=\left|{\lambda }_{c}-{\lambda }_{0}\right|=\left|{\Delta \lambda }_{max}\right|\cdot \frac{{c}^{n}}{{K}^{n}+{c}^{n}}$$

where *c* indicates the cortisol concentration, λ_c_ and λ_0_ denote the resonance wavelength at concentration *c* and the one of the blank, respectively; Δλ_max_ is computed as the difference between the resonance wavelength at saturation value and the blank. Generally, the parameters *n* and *K* denote the Hill fitting constants whereas when n = 1, the Hill model corresponds with the Langmuir model (as in the presented case) and K = 1/K_aff_, where K_aff_ indicates the affinity constant.

Finally, two real seawater samples were collected (in SeaEight Company, Portugal) from two tanks, one with and one without fish. The type of such production is intensive with high biomass (quantity of fish) in each tank. Stocking density of 100 kg/m^3^ (70 m^3^ total volume) in RAS is used for the maintenance of water quality parameters and without, or at least avoid, detrimental effects on fish performance. In the case of real samples, both the samples were diluted with PBS with the following dilution factors: 1:10, 1:100, 1:500, 1:1000, and 1:5000. To further confirm the estimated cortisol concentration, present in the aquaculture water samples, a liquid–liquid extraction was implemented to maximize the response of cortisol in liquid chromatography coupled to tandem mass spectrometry (LC–MS/MS) analysis.

The Liquid–liquid extraction was performed by adding 6 mL of ethyl acetate (extractant solvent) to 6 mL of the aquaculture water sample. The mixture was vortexed for 5 min and centrifuged at 5500 × g for 5 min at 7 °C. The upper phase was collected and evaporated under a moderate nitrogen stream. The residue was redissolved with a water solution (containing 0.5% v/v acetic acid): methanol (50:50 v/v) to a final volume of 1 mL and filtered through Chromafil PTFE 0.2 µm syringe filters (Macherey–Nagel, Düren, Germany). Each sample was extracted twice. Finally, 10 µl of sample extract was injected into the LC–MS/MS system. The sample extracts were first separated on a reverse-phase column using a Symmetry C18 column, maintained at 40 °C. The injection volume was 10 µl. Each sample was injected twice.

### Supplementary Information

Supplementary Information.

## Data Availability

All data generated or analysed during this study are included in this published article [and its supplementary information files], however raw data will be available from the corresponding author on reasonable request.
