# Supplementary material for: POF-based biosensors for cortisol detection in seawater as a tool for aquaculture systems
Source: Sci Rep. 2024 Jun 7;14:13117. doi: 10.1038/s41598-024-63870-7 (PMC11161578; doi:10.1038/s41598-024-63870-7)
Supplement: Supplementary file 1 — Supplementary Information. [file 41598_2024_63870_MOESM1_ESM.docx]

Supplementary Information File

POF-based biosensors for cortisol detection in seawater as a tool for Aquaculture systems

Francesco Arcadio^a^, Simone Soares^b,c^, Jan Nedoma^d^, Dayana Aguiar^e^, Ana Cristina Pereira ^e,f^, Luigi Zeni^a^, Nunzio Cennamo^a*^, Carlos Marques^b,g*^

^a^ University of Campania Luigi Vanvitelli, Department of Engineering, Via Roma 29, 81031, Aversa, Italy

^b^ CICECO – Aveiro Institute of Materials & Physics Department, University of Aveiro, Campus Universitário de Santiago, 3810-193, Aveiro, Portugal

^c^ I3N & Physics Department, University of Aveiro, Campus Universitário de Santiago, 3810-193, Aveiro, Portugal

^d^ Department of Telecommunications, VSB – Technical University of Ostrava, Ostrava, 70800, Czech Republic

^e^ ISOPlexis, Centre for Sustainable Agriculture and Food Technology, University of Madeira, Campus da Penteada, 9020-105 Funchal, Portugal

^f^ Chemical Process Engineering and Forest Products Research Centre, Department of Chemical Engineering, University of Coimbra, Pólo II—Rua Sílvio Lima, 3030-790 Coimbra, Portugal

^g^ Department of Physics, VSB – Technical University of Ostrava, Ostrava, 70800, Czech Republic

* Corresponding authors: carlos.marques@ua.pt; nunzio.cennamo@unicampania.it


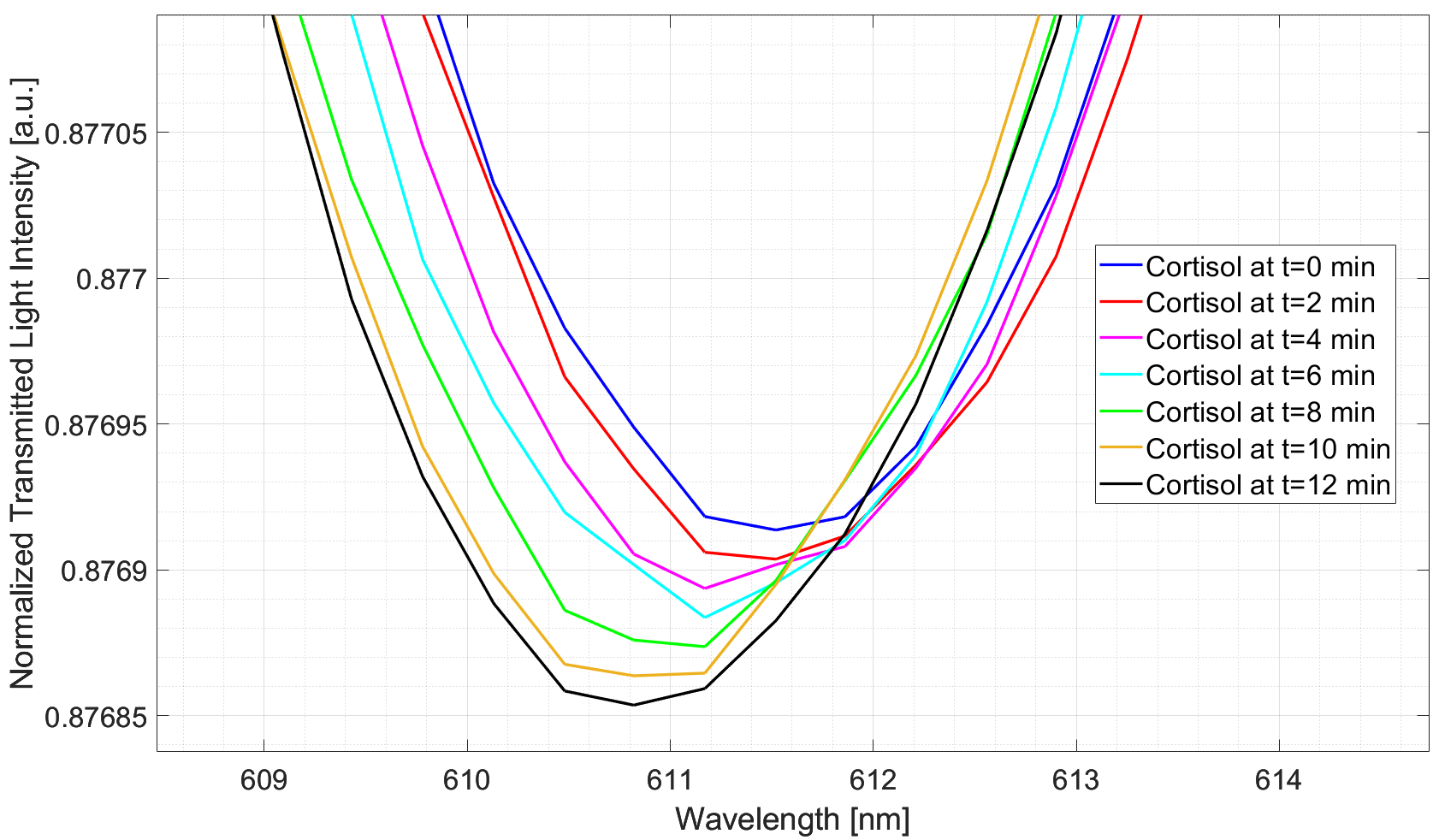


(a)


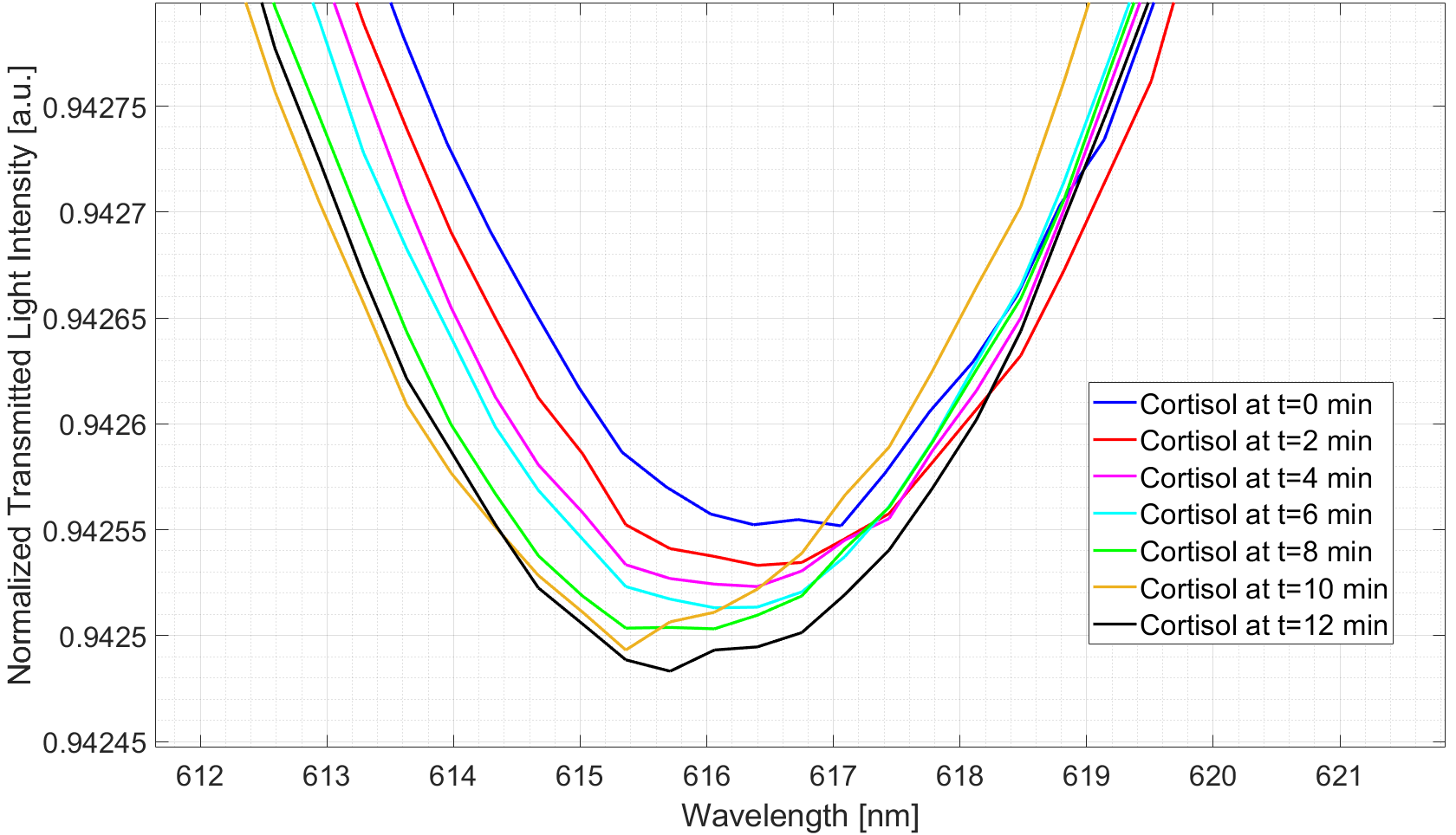


(b)

**Figure S1:** Normalized SPR spectra obtained for cortisol (0.05 ng/mL) at different incubation times on biosensor configurations based on a) lipoic acid and b) cysteamine.
